# PD-L1 expression in EBV-negative diffuse large B-cell lymphoma: clinicopathologic features and prognostic implications

**DOI:** 10.18632/oncotarget.11045

**Published:** 2016-08-04

**Authors:** Wei Xing, Karen Dresser, Rui Zhang, Andrew M. Evens, Hongbo Yu, Bruce A. Woda, Benjamin J. Chen

**Affiliations:** ^1^ Department of Pathology, UMass Memorial Medical Center and University of Massachusetts Medical School, Worcester, MA, USA; ^2^ Department of Biostatistics, Boston University School of Public Health, Boston, MA, USA; ^3^ Division of Hematology/Oncology, Tufts Medical Center, Boston, MA, USA; ^4^ Present Address: Department of Pathology and Laboratory Medicine, VA Boston Healthcare System, West Roxbury, MA, USA

**Keywords:** programmed cell death ligand 1, diffuse large B-cell lymphoma, immune checkpoint, immunotherapy

## Abstract

Programmed cell death ligand 1 (PD-L1) is a cell surface glycoprotein that regulates the cellular immune response and serves as a targetable immune checkpoint molecule. PD-L1 is expressed on tumor cells and the immune microenvironment of several human malignancies, including a subset of aggressive lymphomas. We sought to investigate further the clinical and pathologic features of EBV-negative diffuse large B-cell lymphoma (DLBCL) cases that express PD-L1. Immunohistochemical staining using an anti-PD-L1 monoclonal antibody was performed on DLBCL cases from 86 patients. These patients received standard chemotherapy treatment and were followed for up to 175 months. Overall, 14 cases (16%) were considered positive for PD-L1 in tumor cells. In comparison with PD-L1 negative cases, PD-L1 positive cases had a higher rate of non-GCB type (71% vs. 30%, P=0.0060), and higher Ann Arbor stage (II-IV) (100% vs. 73%, P=0.0327). No significant differences were seen in the immunohistochemical expression of BCL2, MYC, or Ki67. Patients with tumors expressing PD-L1 demonstrated inferior overall survival (OS) upon long term follow up (P=0.0447). Both age/sex-adjusted and multivariate analyses identified PD-L1 as an independent predictor for OS (P=0.0101 and P=0.0424). There was no significant difference, however, in terms of remission rates after first treatment, relapse rates, and progression free survival between the groups. Identification of DLBCL cases that express PD-L1 may serve to select a subset of patients that could further benefit from targeted immunotherapy.

## INTRODUCTION

Immune checkpoint blockade strategies have revolutionized the approach to cancer therapy and have provided oncologists and patients with novel therapeutic options for a range of malignancies [1]. Immunotherapeutic targeting of hematologic malignancies, most notably classical Hodgkin lymphoma, have recently centered on disrupting the programmed death-1 (PD-1) immunomodulatory pathway [2, 3]. Binding of PD-1 by its cognate receptors PD-L1 and PD-L2 inhibits proliferation of activated T cells in peripheral tissues leading to “T-cell exhaustion,” a functional phenotype that can be reversed by PD-1 blockade [4]. Immunohistochemical detection of PD-L1 has been well-documented in a range of human malignancies, including aggressive EBV-positive B-cell lymphomas [5], classical Hodgkin lymphoma [6, 7], melanoma, renal cell carcinoma, and non-small cell lung carcinoma [8]. Interestingly, PD-L1 expression has been observed on tumor cells as well as on non-malignant infiltrating histiocytes, suggesting tumors may elicit an overall immunosuppressive microenvironment as a means of tumorigenesis [5].

These findings have provided a rationale for disrupting the PD-1 axis using antibodies against these antigens with the objective of restoring the anti-tumor activity of suppressed T cells. Clinical trials with humanized monoclonal antibodies against PD-L1 and PD-1 have yielded robust, durable responses in patients with advanced malignancies including metastatic melanoma, tumors with mismatch-repair deficiency, and relapsed or refractory classical Hodgkin lymphoma [9–12]. Responses have also been reported in smaller series of hematologic malignancies, including acute myeloid leukemia, follicular lymphoma, and a number of cases of diffuse large B-cell lymphoma (DLBCL) [13–15]. Notably, in a subset of cases examined, clinical responsiveness to PD-1 blockade correlated with tumor-specific expression of PD-L1 as detected by immunohistochemistry (IHC) [8, 12].

DLBCL, not otherwise specified (NOS), is the most common type of non-Hodgkin lymphoma among adults and is both clinically and pathologically heterogeneous. Several pathologic features are known to predict a worse clinical outcome for DLBCL patients, including non-germinal center B-cell (non-GCB) phenotype [16], increased MYC protein expression [17], and gene rearrangements involving *BCL2*, *BCL6*, and/or *MYC* [18–20]. Identifying additional features that could predict potential response to immunotherapy is important to both understanding the immunomodulatory mechanisms of hematologic malignancies and to developing novel chemotherapeutic regimens.

In the present study, we sought to investigate further the clinical and pathologic features of EBV-negative DLBCL cases with PD-L1 expression. Using a well-annotated cohort of patients with treatment information and long term clinical follow-up, we examined the immunohistochemical expression of PD-L1 in tumor cells and the microenvironment, and correlated these data with additional histologic parameters and clinical outcome data.

## RESULTS

### Pathologic characteristics

**Figure 1:**
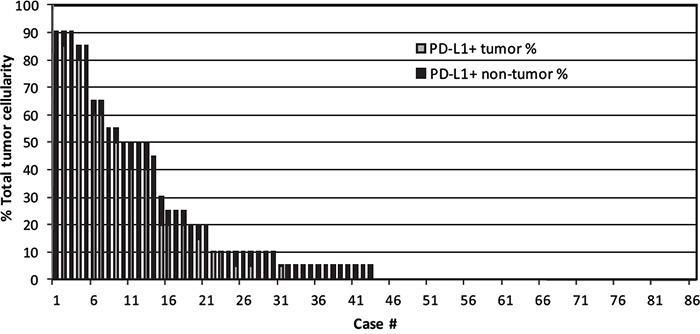
PD-L1 scores in lymphoma cells and microenvironment of 86 DLBCL cases Graphical representation of the percentage of total cells within the tissue section staining positive with anti-PD-L1, and showing the contributions of malignant cells (white bars) and non-malignant cells (black bars) in each case of DLBCL.

**Figure 2:**
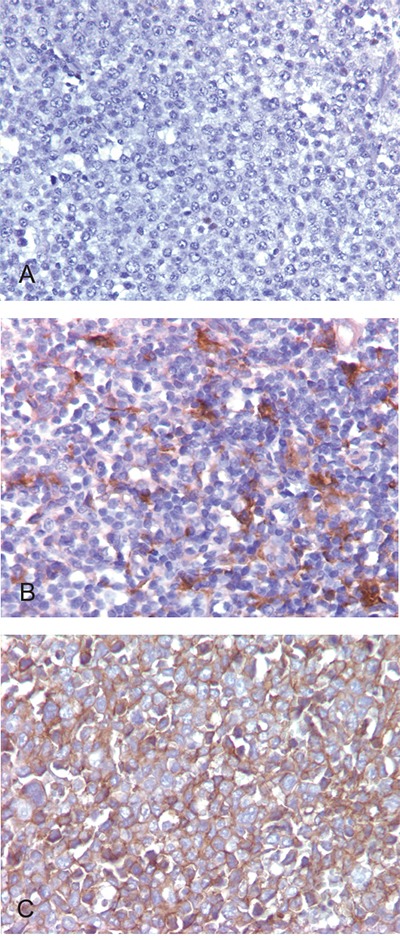
PD-L1 expression detected by immunohistochemical staining in DLBCL **A.** Negative staining. **B.** Tumor cells negative for PD-L1 but histiocytes in the microenvironment staining positive, about 10% of the total cellularity. **C.** Tumor cells showing strong membrane positivity for PD-L1. Original magnification: 400x.

PD-L1-positive tumor cells, along with PD-L1-positive non-malignant cells, were recorded as a percentage of the total cellularity within tumor sections (Figure 1). A threshold of >30% PD-L1-positive tumor cells captured the majority of cases that exhibited positive PD-L1 staining (14/27, 52%), similar to the findings of a recent study [21]. Of the 86 DLBCL cases, 14 cases (16%) were considered PD-L1 positive cases based on this threshold. Of the 72 PD-L1 tumor negative cases, 23 cases (27%) showed at least 5% PD-L1 positive staining in the non-malignant cells (mPD-L1 positive, Figure 1). Representative photographs of DLBCL cases stained with PD-L1 IHC with corresponding scores are shown in Figure 2. Eighty-three cases had evaluable material to be further subcategorized into GCB and non-GCB types according to the cell-of-origin (COO) Hans algorithm [16]. Out of 14 PD-L1 positive cases, 4 cases (29%) were GCB type and 10 cases (71%) were non-GCB type. Out of 69 PD-L1 negative cases, 48 cases (70%) were GCB type and 21 cases (30%) were non-GCB type. PD-L1 positive cases had a higher rate of non-GCB phenotype compared to PD-L1 negative cases (71% vs. 30%, P=0.0060, Table 1).

**Table 1:** Pathologic features of DLBCL cases with PD-L1 expression

Pathologic Features	PD-L1 positive	PD-L1 negative		Total	P^Φ^	P^#^	P^†^
		mPD-L1-positive	mPD-L1-negative				
Phenotype							
GCB	4 (29%)	13 (57%)	35 (76%)	52	0.0060^*^	0.1067	0.0027^*^
Non-GCB	10 (71%)	10 (43%)	11 (24%)	31			
BCL2							
Positive (>30%)	11 (79%)	15 (71%)	24 (51%)	50	0.2283	0.1841	0.1220
Negative	3 (21%)	6 (29%)	23 (49%)	32			
BCL6							
Positive (>30%)	5 (36%)	16 (76%)	32 (70%)	53	0.0146^*^	0.7714	0.0306^*^
Negative	9 (64%)	5 (24%)	14 (30%)	28			
CD10							
Positive	1 (7%)	7 (32%)	23 (49%)	31	0.0134^*^	0.2041	0.0050^*^
Negative	13 (93%)	15 (68%)	24 (51%)	52			
MUM1							
Positive (>30%)	13 (93%)	12 (55%)	21 (48%)	46	0.0029^*^	0.7944	0.0040^*^
Negative	1 (7%)	10 (45%)	23 (52%)	34			
MYC							
< 40%	7 (50%)	11 (48%)	34 (74%)	52	0.3659	0.0590	0.1108
≥ 40%	7 (50%)	12 (52%)	12 (26%)	31			
Ki67							
< 80%	10 (71%)	14 (61%)	34 (71%)	58	0.7789	0.4275	1.0000
≥ 80%	4 (29%)	9 (39%)	14 (29%)	27			

Fisher's exact test.

* P value is significant (<0.05);

PΦ PD-L1-positive versus PD-L1-negative;

P# mPD-L1-positive versus mPD-L1-negative;

P† PD-L1-positive versus mPD-L1-negative.

The overall positivity rates of CD10, BCL2, BCL6, and MUM1 were 36%, 58%, 62%, and 53%, respectively. MYC positive tumor cells by IHC ranged from 0% to 80% (mean ± SD, 28.3±25.3%). The Ki67 labeling index ranged from 10% to 95% (mean±SD, 56.2±26.2%). CD10, BCL6, and MUM1 showed significant differences between the PD-L1 positive and negative groups reflecting the non-GCB vs. GCB phenotype differences (Table 1). When comparing the immunohistochemical expression of BCL2, MYC, or Ki67 between the PD-L1 positive and PD-L1 negative groups, no significant differences were seen (Table 1). Separate analysis of mPD-L1 positive vs mPD-L1 negative cases showed no significant differences in these IHC studies.

### Clinical characteristics

Clinical features including age, gender, presence of B symptoms, pre-treatment LDH, IPI score, and bone marrow involvement showed no statistical difference between PD-L1 positive and negative groups (Table 2). The total extralymphatic organ (organs other than bone marrow, lymph node and spleen) involvement rate was 59% (46/78, Table 2). Both the PD-L1 tumor positive group (10/12, 83%) and mPD-L1 positive group (17/20, 85%) showed greater extralymphatic organ involvement compared to the mPD-L1 negative group (19/46, 41%), which was statistically significant (P=0.0206 and P=0.0012 respectively, Table 2). The rates of high Ann Arbor stage (II-IV) at diagnosis of mPD-L1 negative, mPD-L1 positive and PD-L1 positive cases were 67% (30/45), 86% (18/21) and 100% (13/13), respectively. PD-L1 positive cases had significantly higher initial staging than PD-L1 negative cases (P=0.0327).

**Table 2:** Clinical features of DLBCL cases with PD-L1 expression

Clinical Features	PD-L1 positive	PD-L1 negative		Total	P^Φ^	P^#^	P^†^
		mPD-L1-positive	mPD-L1-negative				
Age (years)							
≤ 60	5 (36%)	5 (22%)	14 (29%)	24	0.5216	0.7749	0.7428
> 60	9 (64%)	18 (78%)	35 (71%)	62			
Gender							
Male	7 (50%)	15 (65%)	18 (37%)	40	0.7793	0.0413^*^	0.5367
Female	7 (50%)	8 (35%)	31 (63%)	46			
B symptoms							
Presence	2 (25%)	3 (18%)	12 (33%)	17	1.0000	0.3332	1.0000
Absence	6 (75%)	14 (82%)	24 (67%)	44			
Pre-treatment LDH							
Normal	2 (20%)	11 (58%)	15 (36%)	28	0.2961	0.1618	0.4668
Elevated	8 (80%)	8 (42%)	27 (64%)	43			
IPI score							
0 - 1	1 (10%)	7 (41%)	13 (30%)	21	0.2621	0.5438	0.2634
2 - 5	9 (90%)	10 (59%)	31 (70%)	50			
Ann Arbor Stage							
I	0 (0%)	3 (14%)	15 (33%)	18	0.0327^*^	0.1423	0.0138^*^
II - IV	13 (100%)	18 (86%)	30 (67%)	51			
BM involvement							
Present	3 (30%)	3 (19%)	2 (6%)	8	0.1309	0.3164	0.0782
Absent	7 (70%)	13 (81%)	30 (94%)	50			
Extralymphatic involvement							
Present	10 (83%)	17 (85%)	19 (41%)	46	0.1083	0.0012^*^	0.0206^*^
Absent	2 (17%)	3 (15%)	27 (59%)	32			
Complete and partial remission after first treatment							
Yes	5 (71%)	18 (86%)	33 (75%)	56	0.6473	0.5200	1.0000
No	2 (29%)	3 (14%)	11 (25%)	16			
Relapse after initial treatment							
Yes	9 (82%)	10 (56%)	31 (69%)	50	0.4861	0.3850	0.4829
No	2 (18%)	8 (44%)	14 (31%)	24			
Outcome							
Dead	9 (82%)	8 (47%)	26 (59%)	43	0.1806	0.5662	0.2932
Alive	2 (18%)	9 (53%)	18 (41%)	29			

Fisher's exact test.

* P value is significant (<0.05);

PΦ PD-L1-positive versus PD-L1-negative;

P# mPD-L1-positive versus mPD-L1-negative;

P† PD-L1-positive versus mPD-L1-negative.

The majority of patients (73/86, 85%) received rituximab, cyclophosphamide, doxorubicin, vincristine and prednisone (R-CHOP) as standard chemotherapy. Other treatment regimens included dose-adjusted etoposide, vincristine, doxorubicin, cyclophosphamide and prednisone (EPOCH); hyper-fractionated cyclophosphamide, vincristine, doxorubicin and dexamethasone (hyper-CVAD); chlorambucil plus prednisone; rituximab alone; methotrexate alone; and radiation alone. None of the patients received hematopoietic stem cell transplant. All patients were followed by clinical and radiological assessments over a period ranging from 2 days to 175 months (median follow-up 21 months).

Of 72 patients who received R-CHOP treatment and had available followup information, the overall response rate (complete and partial remission) was 78% (56/72). No significant differences were found among the three groups (Table 2).

**Figure 3:**
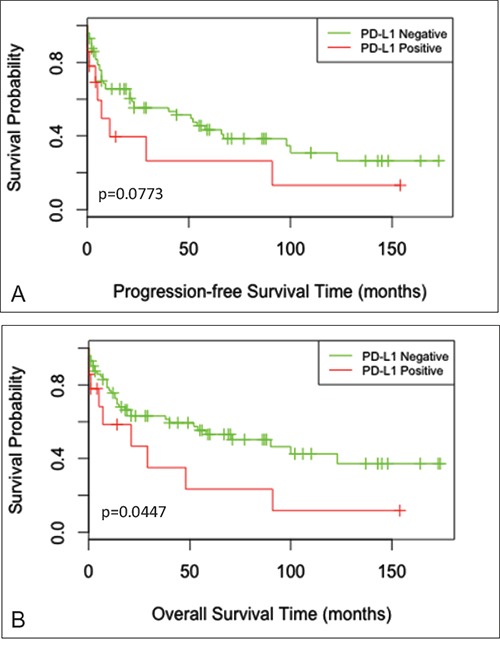
Survival curves for progression-free survival and overall survival Kaplan-Meier curves for **A.** progression-free survival (PFS) time (P=0.0773) and **B.** overall survival (OS) time (P=0.0447) among PD-L1 positive and PD-L1 negative cases.

After initial treatment, the median progression-free survival (PFS) time was 18.5 months (range, 2 days to 173 months) and the median overall survival (OS) time was 21 months (range, 2 days to 174 months). For survival analyses, the 86 cases were divided into 2 groups: PD-L1 positive and PD-L1 negative groups (including mPD-L1 positive and mPD-L1 negative cases). Kaplan-Meier curves of PFS and OS showed apparent decreased survival of the PD-L1 positive group (Figure 3), although only the difference in OS was statistically significant by Log Rank test (P_PFS_=0.0773; P_OS_=0.0447). Within the PD-L1 negative group, there was no significant difference in OS between the mPD-L1+ and mPD-L1- subgroups (data not shown).

Age and sex-adjusted Cox regression analysis of prognostic factors for OS revealed PD-L1 expression (P=0.0101), high stage (II-IV) (P=0.0064), and intermediate-high IPI (2-5) (P=0.0120) were statistically significant predictors of OS (Table 3). Non-GCB type was also an unfavorable factor with borderline statistical significance (P=0.0657). In a multivariate model examining these factors, PD-L1 expression remained a statistically significant factor for OS (P=0.0424) (Table 3).

**Table 3:** Prognostic factors affecting the OS of patients with EBV- DLBCL

Characteristic	Age- and sex-adjusted analysis		Multivariate analysis^#^	
	HR (95% CI)	*P*	HR (95% CI)	*P*
PD-L1+ vs. PD-L1-	2.740 (1.272 – 5.904)	0.0101^*^	2.418 (1.031 – 5.670)	0.0424^*^
Non-GCB vs. GCB	1.824 (0.962 – 3.460)	0.0657	1.321 (0.640 – 2.730)	0.4517
Stage II-IV vs. Stage I	4.318 (1.508 – 12.362)	0.0064^*^	3.125 (0.880 – 11.101)	0.0781
IPI intermediate-high vs IPI low	3.949 (1.353 – 11.523)	0.0120^*^	2.961 (0.823 – 10.658)	0.0967

* P value is significant (<0.05).

# The variables included in multivariate analysis for OS were age, sex, expression of PD-L1, cell of origin, stage, and IPI.

## DISCUSSION

Immune checkpoint blockade therapy is a promising chemotherapeutic strategy that will likely lead to novel multimodal and combinatorial approaches to treating hematologic malignancies [1]. In the case of B-cell non-Hodgkin lymphoma, limited but encouraging results have been observed in early clinical trials [13–15]. In many of the recent clinical trials of anti-PD1/PDL1 immunotherapy, PD-L1 expression in tumor cells was not used as a prerequisite for patient enrollment. Retrospective analyses, however, have shown that PD-L1 positivity in tumor cells, as detected by immunohistochemistry, may predict improved response to anti-PD1/PD-L1 therapy in melanoma, non-small cell lung carcinoma, renal cell carcinoma, and urothelial carcinoma [8, 12, 22]. Thus, profiling tumors for PD-L1 expression may aid in the rational selection of patients for whom immunotherapy may be expected to provide a more robust response.

In this study, we correlated PD-L1 expression in EBV-negative DLBCL cases with clinical and pathologic features. In our cohort, 16% of cases were positive for PD-L1 in tumor cells and an additional 27% had positive microenvironment staining. Several recent studies have examined the incidence of PD-L1 positivity in DLBCL, NOS cases and variants of DLBCL, including T-cell/histiocyte-rich large B-cell lymphoma and EBV-positive DLBCL of the elderly [5, 21, 23–25]. The proportion of DLBCL, NOS cases in our study that was positive for PD-L1 was comparable to that seen in these previous studies examining similar cohorts of DLBCL cases. Furthermore, we confirm previous observations that PD-L1 positive DLBCL cases were more commonly of the non-germinal center B-cell type, which typically portends a poorer overall prognosis. In addition, PD-L1 expression was not significantly associated with other prognostic features such as BCL2 or Ki67 proliferation index.

In addition to non-GCB phenotype, we found that patients with PD-L1 positive DLBCL had higher-stage tumors than those with PD-L1 negative DLBCL. We also found a trend towards poorer outcomes in terms of PFS and OS for patients with PD-L1 positive tumors. Decreased OS was found to be statistically significant in our cohort. Two analyses of clinical outcome in PD-L1 positive DLBCL have recently been reported [21, 25]. Kiyasu, et al [21], examined a cohort of 1253 DLBCL cases and found that, in a smaller subset of cases with available clinical data (273 cases), PD-L1 positivity in tumor cells correlated with poorer overall survival. This cohort included cases of DLBCL, NOS, as well as variants such as EBV+ DLBCL. In this study, a threshold of >30% of tumor cells positive for PD-L1 was identified based upon the observation that the majority of cases were captured by this analysis. In the current study, we find that a similar threshold appears to identify a cohort of DLBCL cases that also have poorer overall survival providing an important external validation of the prior findings and supporting the use of this threshold. Another recent study by Kwon, et al [25], examined 126 DLBCL cases. This cohort included cases of DLBCL, NOS, as well as EBV+ DLBCL cases. In this study, a threshold of 10% PD-L1 positivity was used, yielding about 30% of DLBCL cases with at least moderate staining. No significant correlation with patient outcome was seen in this analysis.

Among the studies examining PD-L1 expression in DLBCL, it should be noted that the thresholds for determining PD-L1 positivity have varied widely. In earlier studies of PD-L1 staining in DLBCL, a lower threshold, such as 5% tumor positivity, was employed [5], similar to that used for the early studies of PD-L1 expression in melanoma [8]. In terms of predicting prognosis, it seems that higher expression levels, such as 30%, as seen in the study by Kiyasu, et al, and our study, may be more relevant to identifying patients at risk of poorer clinical outcome. In our study, we found that lowering the cutoff threshold to 5, 10, or 20% resulted in non-statistically significant survival differences (data not shown). The study by Kwon, et al, further highlights the potentially tenuous relationship between PD-L1 expression and prognosis in DLBCL. A limitation to any proposed cutoff value for PD-L1 positivity is the inherently subjective nature of evaluating immunohistochemical staining. Computer aided detection and double-staining techniques may assist in the evaluation, but these approaches may not be practical for routine clinical use and carry their own limitations. Further complicating these analyses is the heterogeneous nature of the DLBCL, NOS category, and the inclusion of variants such as EBV+ cases in the cohorts examined in the studies by Kiyasu, et al, and Kwon, et al. In our study, we have limited our cohort to EBV-negative DLBCL in an effort to exclude the influence of EBV positivity on prognosis. Nonetheless, several studies have found that PD-L1 expression in a wide range of different solid tumor types, including breast [26], bladder [27], stomach [28], and non-small cell lung carcinoma [29] correlates with poorer prognosis [30]. Clearly, better-powered studies involving larger patient populations receiving standardized chemotherapy regimens and long-term followup are needed to build upon these compelling findings.

The finding that a subset of DLBCL cases had PD-L1 expression in non-malignant cells within the tumor microenvironment supports previous studies that observed a distinct proportion of classical Hodgkin lymphoma and DLBCL with this finding [5]. In our cohort, this finding did not translate into a significant prognostic factor, as was found by Kiyasu, et al [21]. The mechanisms responsible for the upregulation of PD-L1 in the tumor microenvironment need further investigation, but are an intriguing source of tumor-induced immunomodulation that could potentially be targeted.

In the case of classical Hodgkin lymphoma, at least two independent genetic mechanisms, 9p24 amplification and EBV infection, are thought to lead to overexpression of PD-L1 in the malignant Reed-Sternberg cells [6, 7]. Similarly, the majority of EBV-positive aggressive B-cell lymphomas, including EBV-positive DLBCL, show upregulation of PD-L1 [5]. For DLBCL-NOS, a recent study identified a genetic basis of PD-L1 overexpression through translocations between the *PD-L1* and *IGH* gene loci [31]. These cases may represent a distinct subtype of DLBCL that will require further characterization.

With the anticipation of additional clinical trials of immunotherapy directed at the PD-1 axis, treatment challenges will include determining the ideal time to initiate targeted immunotherapy; whether to combine immunotherapy with conventional chemotherapy, other immune checkpoint inhibitors, or hematopoietic stem cell transplant; and whether treatment should be continued following the first remission to prevent recurrence. Identification of DLBCL cases that express PD-L1 may form a rational basis for guiding therapy and can serve to select a subset of patients that could further benefit from targeted immunotherapy. Threshold levels of PD-L1 expression will need to be further examined to determine a biologically relevant level of expression that can predict tumor response to therapy and/or predict patient prognosis.

## MATERIALS AND METHODS

### Case selection

Cases of DLBCL, NOS, diagnosed between 2000 and 2014 were retrieved from the surgical pathology files and medical records of our institution. The study was approved by the University of Massachusetts Medical School Institutional Review Board. All DLBCLs were diagnosed and classified according to 2008 World Health Organization (WHO) criteria. Eighty-six DLBCL cases were included, composed of 46 female and 40 male patients, with a median age of 70 years (range 15-91 years). The primary sites involved by DLBCL in descending order of frequency included lymph node (32 cases), soft tissue (18 cases), spleen (5 cases), bone (4 cases), central nervous system (4 cases), skin (4 cases), salivary gland (3 cases), paranasal sinuses (3 cases), small bowel (3 cases), testis (3 cases), breast (2 cases), liver (2 cases), lung (2 cases), and bladder (1 case). All cases were negative for EBV-encoded RNA (EBER) by in situ hybridization study.

### Immunohistochemistry and evaluation

Immunohistochemistry using a rabbit anti-PD-L1 monoclonal antibody (clone E1L3N, #13684, Cell Signaling, Danvers, Massachusetts) was performed on 5 μm-thick, formalin-fixed paraffin embedded (FFPE) tissue sections and tissue microarray sections using a Dako Autostainer (Dako Corporation, Carpinteria, CA) with antigen retrieval methods (0.01M citrate buffer at pH 6.0) as described previously [5]. The UltraView Universal DAB Detection kit (#760-500, Ventana Medical Systems, Tuscon, AZ) was used according to the manufacturer instructions. Counterstaining was done as part of the automated staining protocol using hematoxylin (#760-2021, Ventana Medical Systems).

All IHC-stained sections were evaluated and scored by two hematopathologists independently. Discrepancies in scoring (<10% of cases) were resolved by consensus conference between the two pathologists. Threshold values of 30% for BCL2, BCL6, CD10, and MUM1 staining were chosen reflecting routine clinical practice. MYC and Ki67 expression was recorded using a percentage scale of positive tumor ranging from 0% to 100%. Staining intensity of PD-L1 was scored as follows: 0 (no staining), 1+ (weak), 2+ (moderate), or 3+ (strong). Tumor cells exhibiting 2+ or 3+ membrane staining were recorded as a percentage of total tumor cellularity. PD-L1-positive non-malignant cells as a percentage of total tumor cellularity was also recorded. Appropriate external positive (placenta) and negative (tonsil) controls were included with each staining run.

### Clinical data

Patient demographics, clinical data, treatment and outcome information were obtained from the medical record. Clinical parameters included: presence of B symptoms, performance status, CBC, kidney and liver function tests, lactate dehydrogenase (LDH), bone marrow involvement, other extranodal sites involvement, bulky disease (>10cm), Ann Arbor stage, International Prognostic Index (IPI), positron emission tomography-computed tomography (PET/CT) scan at diagnosis and after treatment, treatment history, response to first treatment (complete remission, partial remission, stable disease and progressive disease), major complications to treatment (e.g., neutropenic fever, sepsis, chronic heart failure, renal failure, etc), the dates of disease progression, relapse or death and cause of death.

### Statistical analysis

Comparisons of clinical data between groups were carried out using the Fisher exact test, Chi-Square test, and survival analysis in SAS v9.3 (SAS Software, Cary, NC, USA) and R 3.2.1. Progression-free survival (PFS) was measured from the date of original diagnosis to the date of last follow-up or of the first progression, relapse or death, toxicity events being excluded. Overall survival (OS) was measured from the date of original diagnosis to the date of last follow-up or death from any cause. PFS and OS were estimated using the Kaplan-Meier method. Differences of PFS or OS at different PD-L1 expression levels were assessed using the Log-Rank tests at the two-sided significance level of 0.05.

Age and sex-adjusted and multivariate analyses were conducted to evaluate the potential association between PD-L1 expression as well as other covariates with clinical outcomes. The stratified Cox proportional hazards model was used to determine hazard ratios and confidence intervals (CI) at the 95th confidence level. The variables included in multivariate analysis for OS were age, sex, expression of PD-L1, GCB/non-GCB type, stage and IPI. All of these variables had a P-value <0.1 in the age- and sex-adjusted analysis. Occasional missing values in the data set did not appear to present significant problems in the modeling strategy. The proportional hazard assumptions were satisfied in both age and sex-adjusted and multivariate analyses based on the log-log plots and Schoenfeld residuals method.
